# Broadband mid-infrared amplified spontaneous emission from Er/Dy co-doped fluoride fiber with a simple diode-pumped configuration

**DOI:** 10.1038/s41598-021-84950-y

**Published:** 2021-03-08

**Authors:** Kenji Goya, Akira Mori, Shigeki Tokita, Ryo Yasuhara, Tetsuo Kishi, Yoshiaki Nishijima, Setsuhisa Tanabe, Hiyori Uehara

**Affiliations:** 1 Faculty of Systems Science and Technology, Akita Prefectural University, 84-4 Ebinokuchi, Tsuchiya, Yurihonjo, Akita Japan; 2 National Institutes of Natural Sciences, National Institute for Fusion Science, 322-6 Oroshi-cho, Toki, Gifu 509-5292 Japan; 3 Institute of Laser Engineering, Osaka University, 2-6 Yamada-oka, Suita, Osaka Japan; 4 Kyoto University, Sakyo-ku, Kyoto 606-8501 Japan; 5 Tokyo Institute of Technology, 2-12-1 Ookayama, Meguro-ku, Tokyo 152-8550 Japan; 6 Yokohama National University, 79-5 Tokiwadai, Hodogaya-ku, Yokohama, 240-8501 Japan

**Keywords:** Optics and photonics, Optical physics, Electrical and electronic engineering

## Abstract

Er^3+^/Dy^3+^ co-doped double-clad ZBLAN optical fiber has been used to obtain amplified spontaneous emission (ASE) broadband light sources cladding-pumped by 980-nm multimode laser diode (LD) sources. It has been demonstrated that mid-infrared broadband emission extending from 2515 to 3735 nm was obtained by energy transfer between Er^3+^ and Dy^3+^. We experimentally investigated the optimum design of Er^3+^/Dy^3+^ co-doped ZBLAN fiber in terms of ion concentration, fiber length, pumping configuration, and pumping power. The ASE output power was more than 2.5 mW when the LD pump power was set at 5 W. To assess its potential for gas sensing applications, the fabricated ASE light source was used to successfully detect methane gas with concentrations at 1% and 5%. The simple and stable construction of our ASE light source is suitable for practical purposes.

## Introduction

Broadband mid-infrared (mid-IR) light sources have been studied as a promising tool for sensing, spectroscopy, and imaging, because various molecules have strong rovibrational absorption lines in this mid-IR region. Fiber lasers, compared to other laser platforms, have many distinctive advantages, such as simplicity, compactness, good beam quality, and ease of heat dissipation. For obtaining mid-IR emissions, ZBLAN (ZrF_4_-BaF_2_-LaF_3_-AlF_3_-NaF) glasses/fibers have been used as the host material for rare-earth-doped gain media due to their low phonon energy and relatively low optical loss. To date, such ZBLAN fibers doped with erbium have been applied in lasers with a wavelength of 2.7–2.9 µm, where versatile high-power fiber lasers have been demonstrated, including all-fiber operation in master oscillator power amplifier systems^1–5^, and wavelength tunable lasers^6,7^.

Fiber lasers with longer wavelengths using Er^3+^:ZBLAN fiber, around 3.5 µm, have been demonstrated by means of dual-wavelength pumping, resulting in 3.55-µm^8^ and 3.3- to 3.8-µm emission^9^. However, a dysprosium doping gain medium is a promising candidate for covering the wavelength region between 2.7 and 3.5 µm^10^, due to the Dy^3+^:^6^H_13/2_ to ^6^H_15/2_ transition^11^ and its broadband gain. Especially for sensing and spectroscopic applications, light emission from Dy^3+^-doped media is comparatively favorable for the emission from Er^3+^-doped media because wavelengths longer than 3 µm show less atmospheric absorption. Such optical windows around 3 µm can be used for sensing and spectroscopic applications in free space and fiber optics. In recent years, the development of Dy^3+^:ZBLAN lasers has been demonstrated^10,12,13^, where a dysprosium fiber laser is used by employing an erbium fiber laser pump. A watt-level laser at 3.15 µm with 73% slope efficiency by means of core-guided pumping^12^, and a 10-W fiber laser at 3.24 µm consisting of an in-house all-fiber system^13^ have been achieved. However, a 2.8-µm pump laser has a significant absorption loss from the polymer cladding, which is the reason they employed core-guided pump/all fiber systems. In general, the core-guided pumping system is delicate, which limits the power, especially in the case of single transverse-mode operation with soft glass fibers such as fluorides because of thermal and mechanical stresses. This is the case even when protective treatments are applied, such as an end cap for cleaved fiber ends and a cooling apparatus for fiber waveguides. While the all-fiber system indispensably requires core-to-core fusion splicing between optical fibers for the pump source and signal laser, a fusion splicing technique that maintains low core propagation losses is not commercially available. In mid-IR source without considering efficient light coupling into optical fibers, photoluminescence from Pr^3+^/Dy^3+^ co-doped selenide-chalcogenide multimode fiber would be useful, where the light source emits maximum output power of 1 mW within the spectral region 3.5–8 µm under laser diodes operating at 1.470 µm, 1.511 µm and 1.690 µm^14,15^.

For the case of directly pumping Dy^3+^ ions, it is difficult to use common commercial high-power laser diodes (LDs) as a pump source due to an inefficient absorption under the wavelengths around 790, 810, 910, and 980 nm. In contrast, erbium can easily be pumped by commercially available 980-nm LDs, which has been indispensable for developing high-power light sources^3,5^ and can also be an efficient sensitizer of Dy^3+^ ions^16^. A few investigations of Er^3+^/Dy^3+^ co-doped glasses have been reported. Co-doped fluoroaluminate bulk glass has been used to obtain broadband emission when pumped by 808- and 980-nm LDs^17^, resulting in high energy transfer efficiencies of Er^3+^:^4^I_13/2_ to Dy^3+^:^6^H_11/2_ (90.3%) and Er^3+^:^4^I_11/2_ to Dy^3+^:^6^H_5/2_ (85.1%) and spontaneous emission at wavelengths from 2500 to 3100 nm, under a 980-nm pump^18^.

In this work, Er^3+^/Dy^3+^ co-doped ZBLAN fiber was used to obtain ~ 3-µm amplified spontaneous emission (ASE) broadband light sources cladding-pumped by 980-nm multimode diode sources. To our knowledge, this is the first report of a mid-IR broadband emission extending from 2515 to 3735 nm with a full-width at half maximum of 1220 nm obtained by energy transfer between Er^3+^/Dy^3+^ co-doped in ZBLAN fiber under a 980-nm pump. We experimentally investigated the optimum design of an Er^3+^/Dy^3+^ co-doped ZBLAN fiber in terms of ion concentration, fiber length, pumping configuration, and pumping power. The fiber diameters were theoretically and empirically determined so that signal light passing through the active fiber was linearly polarized single-mode (LP_01_). The ASE output power was measured as a function of the LD input power, resulting in more than 2.5 mW of output power when the LD pump power was set at 5 W. CH_4_ gas was measured using the fabricated ASE light source to assess the potential for gas sensing applications. Our ASE light source is simply and stably constructed compared with super-continuum light sources and wavelength conversion systems.

## Experiments and results

### Optimization of ion concentration in ZBLAN fiber co-dopant

**Figure 1:**
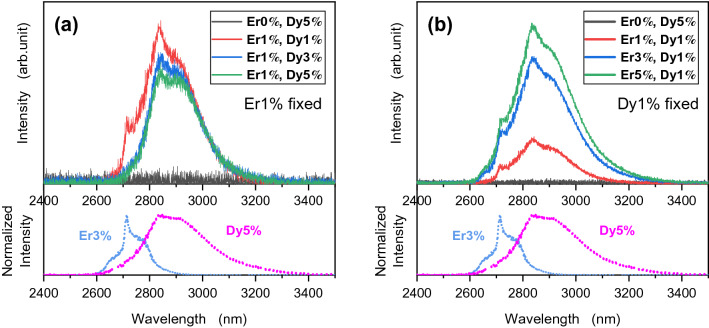
Fluorescence spectrum showing the normalized intensity as a function of wavelength, as obtained from the co-doped bulk media. The emission spectra of Er^3+^:ZBLAN (976-nm pump) in blue and Dy^3+^:ZBLAN (1700-nm pump) in pink are displayed in the lower plots. (**a**) Er^3+^ concentration fixed at 1 mol%. (**b**) Dy^3+^ concentration fixed at 1 mol%. In the legend, a concentration of, for example, 0 mol% Er^3+^ and 5 mol% Dy^3+^ is represented as “Er0%, Dy5%.”

The co-dopant concentration was experimentally determined by measuring the fluorescence from fluoride bulk media with various combinations of Er^3+^/Dy^3+^ concentration ratios, as shown in Fig. 1. Each spectrum was obtained by pumping the fiber with a 976-nm LD source. To compare the obtained spectra against the fluorescent spectra of erbium and dysprosium, the emission spectra of Er^3+^:ZBLAN (976-nm pump) and Dy^3+^:ZBLAN (1700-nm pump) are displayed in the lower graph of each panel. By co-doping with erbium, a dysprosium-derived fluorescence longer than 2.9 µm can be obtained due to the energy transfer from erbium to dysprosium. Meanwhile, the erbium-free ZBLAN glass (black line) produces no emission. As can be seen from Fig. 1a, when the erbium (donor) concentration was fixed, a lower dysprosium concentration exhibited a higher emission intensity due to the concentration quenching and deficiency of donor ions. However, the fluorescence intensity should be greater when the dysprosium (acceptor) concentration is increased to produce a donor-rich condition. The spectral shapes of the 3% and 5% Dy^3+^ samples without the shoulder derived from Er^3+^ also reflected that the doping concentration of 1 mol% Er^3+^ was donor-poor. In Fig. 1b, which shows the fixed dysprosium (acceptor) concentration, even though the intensity increases when the donor concentration is between 3 and 5%, the donor concentration pushes the fluorescence intensity up to almost saturation. Comparison of Fig. 1a,b shows that the combinations “Er3, Dy1” and “Er5, Dy1” show the highest intensity, and could be a candidate for having a higher stimulated emission cross section. Based on these results, the combination of 3 mol% Er^3+^ and 1 mol% Dy^3+^ was chosen as the amplification media, considering that a higher doping concentration tends to produce crystallization and quench the concentration.

### ASE light source

The fiber used in this study was specially purchased from FiberLabs, Inc. The double-clad fiber had a ZBLAN core co-doped with 3 mol% Er and 1 mol% Dy, an undoped circular cladding, and a polyacrylate coating, which functioned as the second cladding. The rare earth elements were doped only in the fiber core to achieve single transverse mode emission resulting in efficient ASE oscillation. The core, first cladding, and second cladding were 15, 200, and 400 μm in diameter, respectively, and the core and first cladding had a numerical aperture of 0.12 and 0.50, respectively, with a cut-off wavelength of 3.2 µm (V-number of 2.51 at 3.0 µm). The fiber was prepared so that both ends had facets angle cleaved at ~ 5° to reduce back-reflection from the fiber end facets and provide effective suppression of the parasitic lasing.

**Figure 2:**
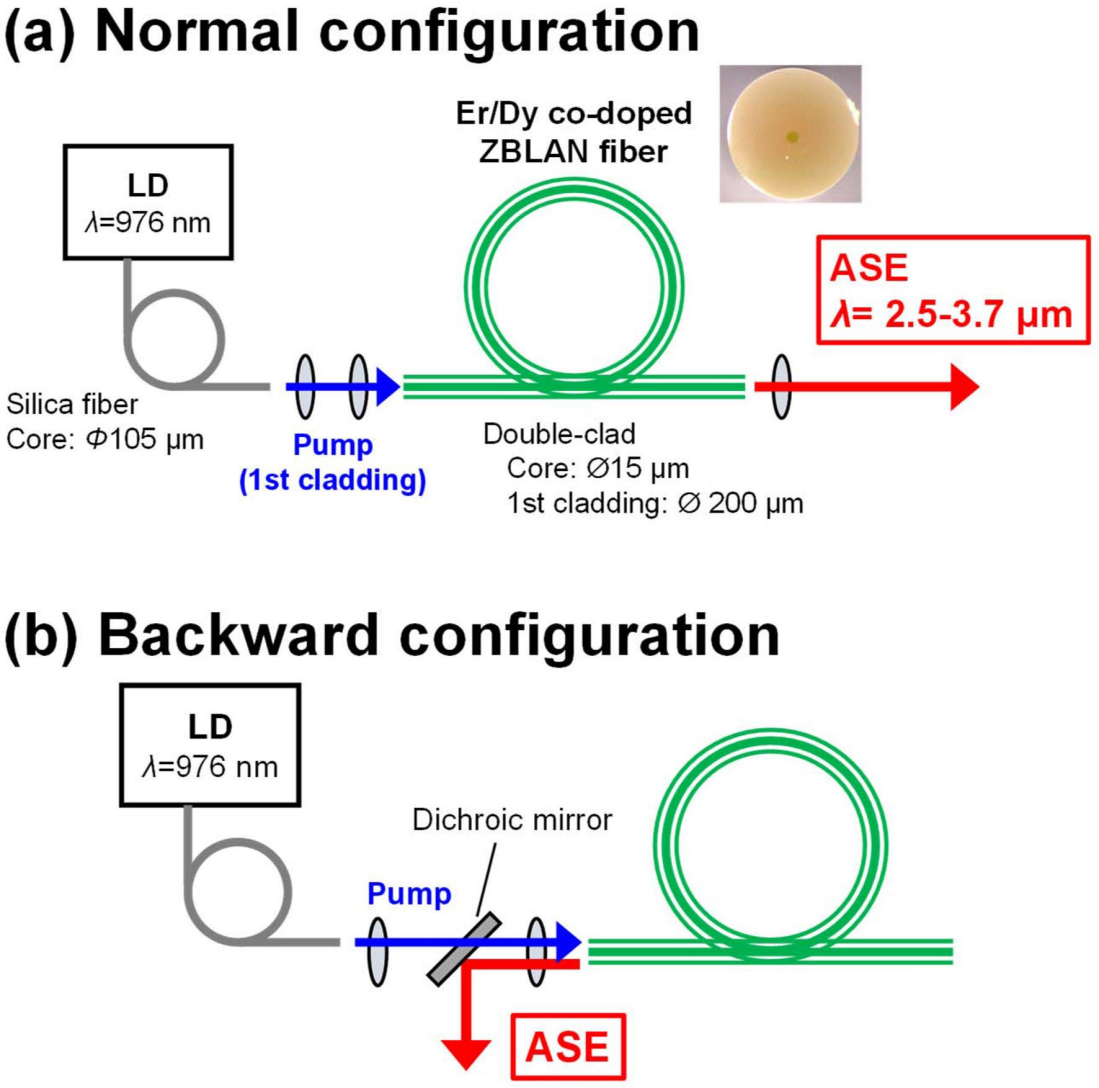
Experimental setup for mid-IR ASE light source. Er^3+^/Dy^3+^ co-doped ZBLAN fiber is pumped by a 980-nm multimode diode source. (**a**) Normal configuration; (**b**) backward configuration.

**Figure 3:**
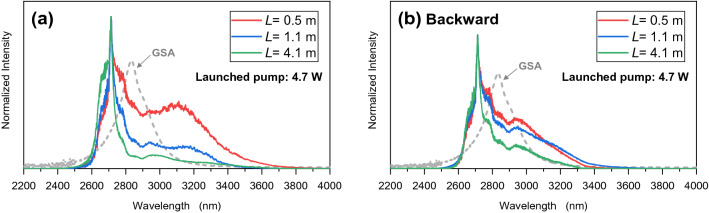
Normalized spectra of ASE from the co-doped ZBLAN fiber in (**a**) forward-pumping configuration and (**b**) backward-pumping configuration.

Figure 2 shows the fabricated ASE light source in (a) forward-pumping configuration with simple and compact form and (b) backward-pumping configuration. The fiber ends were protected by a water-cooled fiber mount to reduce thermal effects. The inset picture is a cross-sectional view of the active fiber. The laser beam of the pump LD with a 105-µm diameter was easily coupled to the 200-µm cladding of the active fiber through a train of collimating optics with focal lengths of 15 and 20 mm. In this experiment, the spectra of the ASE were measured by an optical spectrum analyzer (OSA205C, Thorlabs). Figure 3a shows the normalized emission spectra of the ASE from the source with varying fiber lengths between 0.5 and 4.1 m and launched pump power of 4.7 W. The Dy^3+^ ground-state absorption (GSA), where Er^3+^ ions have no absorption at that band, is also displayed by the gray dashed line. Compared to the emission spectrum shown in Fig. 1, the spectrum shape obtained from the fiber was clearly different, and a comparatively sharp intensity peak appeared around 2700 nm due to the erbium-derived ASE, which does not contribute to energy transfer into dysprosium. It can therefore be said that the erbium- and dysprosium-derived ASE spectra are mixed in the spectra of Fig. 3. In contrast with Dy^3+^, Er^3+^ has a narrower gain bandwidth and much longer lifetime^16^ and hence has a greater gain coefficient. As can be seen, the ASE intensity clearly decreased as the fiber length increased, especially in the longer wavelength region, because the emitted light passing through the fiber was reabsorbed by unexcited Dy^3+^ ions and ASE amplification may have been insufficient after the pump absorption length. Furthermore, for the longer wavelength region around 3 µm, the reabsorption loss in the forward configuration seems to be significant compared to the loss of the backward configuration. By using the shortest fiber length (0.5 m), sufficient amplification due to ASE results in greater intensity in the longer wavelength region.

To investigate the pump absorption, we measured the power of the pump laser after it passed through doped fibers with lengths of 0.5, 1.1, and 4.1 m. The absorption coefficient at 976 nm then was estimated to be about 2.0 dB/m. At a pump power of 5.2 W, the power density was approximately $6 \times 10^{3} {\text{ W}}/{\text{cm}}^{2}$ at the exit end of the fiber, as calculated from the transmittances of 36% corresponding to the remaining power of 1.9 W for the case of fiber length ***L*** of 0.5 m. From the calculation, 0.5-m fibers can be pumped to saturation, which makes it easy to create an inverted distribution in an active fiber while maintaining the gain to some extent. Backward pumping was measured for comparison, as shown in Fig. 3b, in which a dichroic mirror was placed between the lenses on the left side of Fig. 2. The dependency on fiber length is comparatively less than in the case of the forward configuration. Comparison between the spectra in red shows that the normalized intensity at the longer wavelength (> 2.9 µm) of the backward pumping is lower because Er^3+^ fluorescence and amplification were dominant. Based on the results for fiber length and configuration, the subsequent experimental setup for ASE was arranged in the forward pumping configuration with a fiber length of 0.5 m.

**Figure 4:**
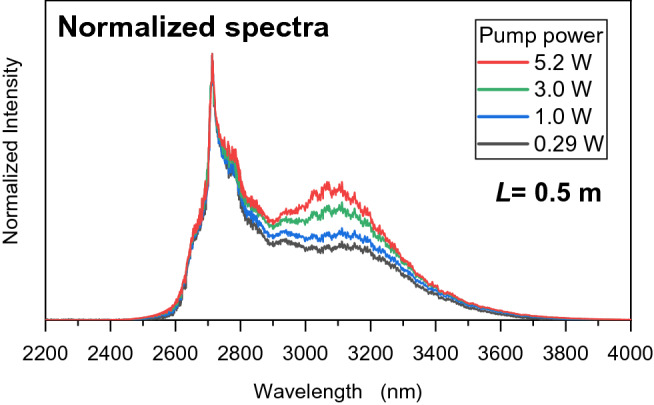
Normalized spectra of ASE with various pump power levels and a fiber length of 0.5 m.

**Figure 5:**
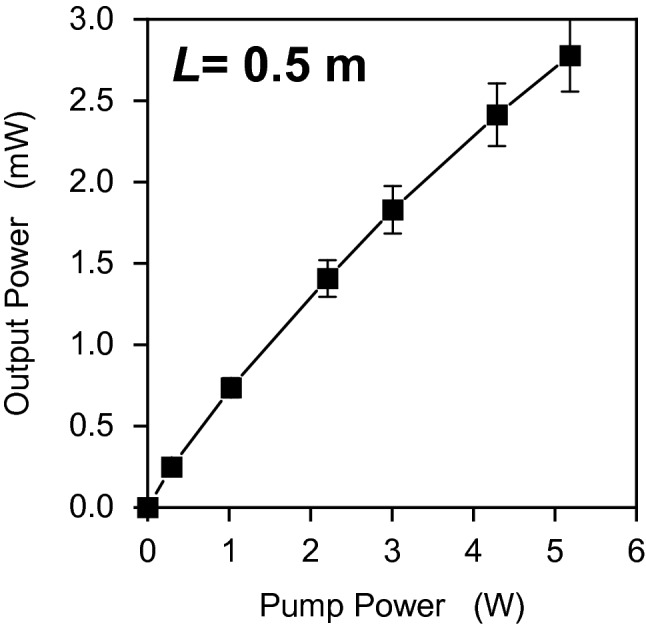
Output power of ASE source as a function of pump power.

To determine the amplification characteristics, the spectra and output power of ASE were observed for various pump powers with a fixed fiber length of 0.5 m, as shown in Figs. 4 and 5. Figure 4 shows the normalized spectra of ASE, where the increasing pump power causes the emission spectrum to reform at a longer wavelength of around 3.1 µm. Such shape reforming was evidently observed as a gain-dependent ASE. The greater amplification at the longer wavelength region was caused by the ASE gain at the longer wavelength being greater than the gain at the shorter wavelength. It can also be found that narrow-band lasing-like spikes appear over the range of 3.0–3.2 µm because of the greater amplification in the longer wavelength region. Even though the spectra obtained in this experiment were stable, it can be inferred that parasitic lasing will eventually happen. Figure 5 shows ASE output power as a function of pump power. The ASE output power reached 2.5 mW when the pump power was 5.2 W. The output ASE power increased in proportion to increasing pump power. The pump power was restricted to approximately 5 W to avoid damage to the fiber end, which had no protective treatment^19,20^.

**Figure 6:**
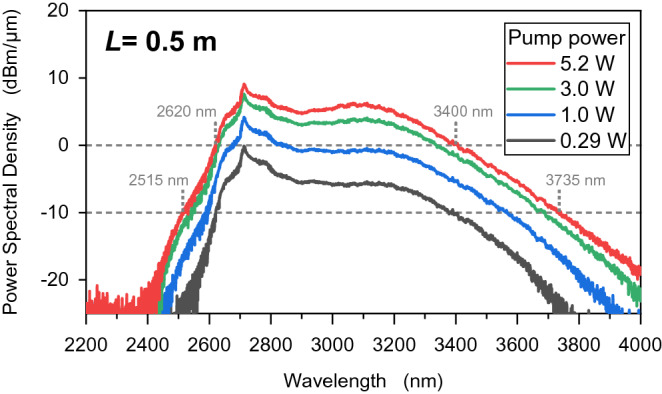
Power spectral density obtained using the measured output power. The spectral band width was measured at power spectral densities of 0 and − 10 dBm/µm.

In Fig. 6, the power spectral density of ASE is represented by the measured output power. The band width was notably broadened by increasing the pump power. The spectral band widths were found to be 780 nm (2620–3400 nm) and 1220 nm (2515–3735 nm) at the power spectral densities of 0 and − 10 dBm/µm, respectively. We focus on the spectral peak at 2.71 and 3.07 µm as the output power changed from 0.29 to 5.2 W. The change in the power spectral density at 3.07 µm was greater than the density at 2.71 µm, where the densities are altered, respectively, by 9.2 dBm/µm and 12.1 dBm/µm. The difference between 2.71 and 3.07 µm proves that Dy^3+^ ions provide a greater gain when the pump power is increased.

**Figure 7:**
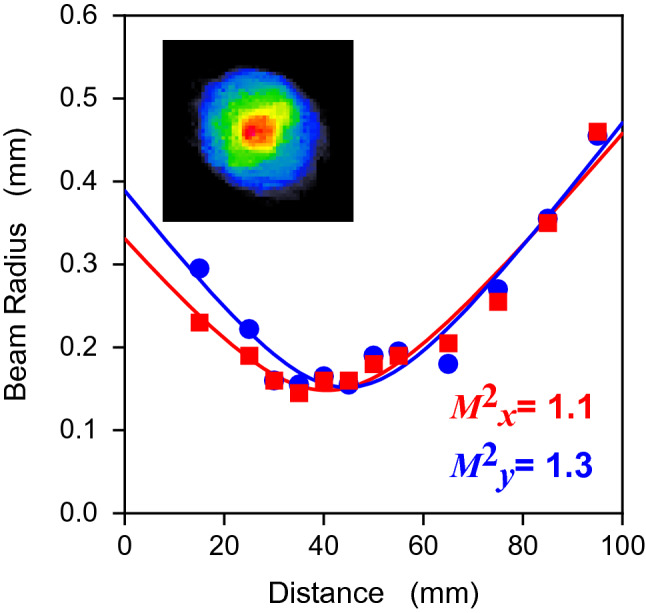
Beam propagation and intensity profile (far field).

The beam radius of the ASE emission was measured with a microbolometer-based IR camera, as a function of the beam propagation position given in Fig. 7. The output transverse beam profile is shown in the inset in the figure. The beam quality factor with $M_{x}^{2}$ of 1.1 and $M_{y}^{2}$ of 1.3 confirmed the beam as being approximately Gaussian. Due to the fiber waveguide with a cut-off wavelength of 3.2 µm, the beam quality was sufficient and favorable for sensing applications.

### CH_4_ gas detection

**Figure 8:**
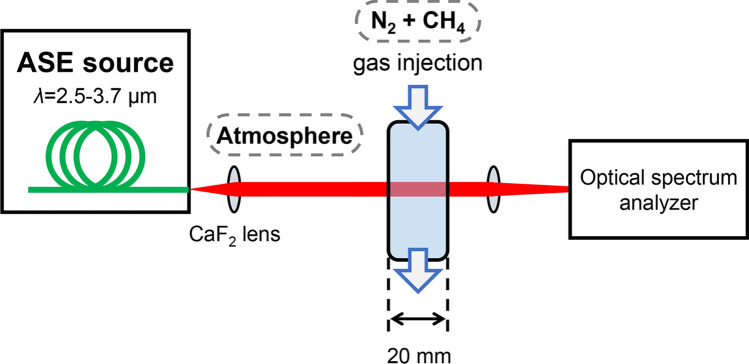
Experimental setup for CH_4_ gas detection.

**Figure 9:**
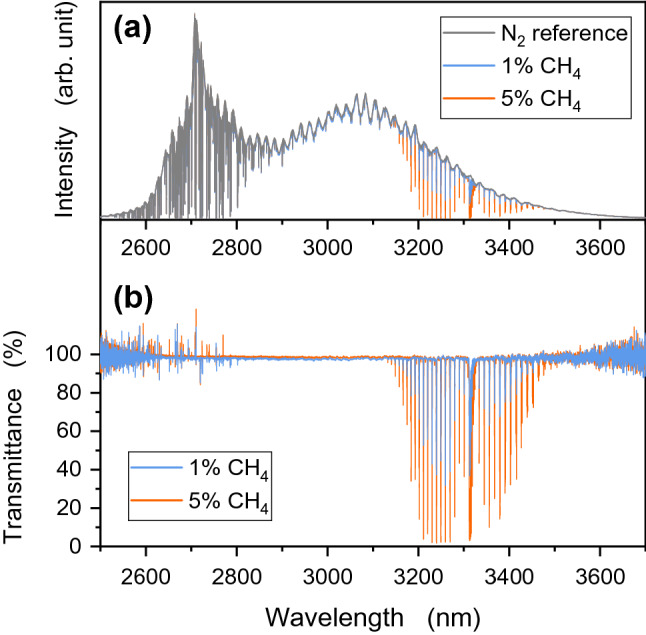
(**a**) Normalized intensity as a function of wavelength. (**b**) Transmission spectrum at each concentration.

To assess its potential for gas sensing applications, a CH_4_ gas detection experiment was performed with the fabricated ASE light source in free-space propagation (Fig. 8). The concentration dependency was tested with two CH_4_ gas concentrations of 1% and 5% mixed with N_2_. The prepared CH_4_ gas was injected into a chamber (20 mm × 40 mm wide and 50 mm tall) equipped with a silica glass optical window. The beam diameter of the ASE signal propagating through the chamber was less than 10 mm. The chamber had silica glass windows with a thickness of 100 µm at the inlet and outlet. The windows caused spectral interference over the entire wavelength region, as shown in Fig. 9a. Such interference ripples were canceled by calculating the transmission loss, where the transmission spectra were simply obtained as a function of wavelength compared with the transmission level obtained when the chamber was filled with pure N_2_ gas, as shown in Fig. 9b.

In the experiment, the ASE light source was stable and all absorption lines derived from C–H stretching vibration in the wavelength region of 3.1–3.5 µm were successfully observed for each concentration. Many absorption lines less than 2.9 µm, which were derived from atmospheric water vapor, are clearly observed in Fig. 9 because the signal beam was propagating in the atmosphere, as can be seen from the experimental setup (Fig. 8). Due to the source’s broad band of 2.5–3.7 µm, species other than CH_4_, such as water vapor, NH_3_, CH_2_O, hydrocarbons, and nitrogen oxides, can be interrogated. In the wavelength, discrimination between other straight-chain alkanes C_n_H_2n+2_ (CH_4_ and C_2_H_6_) or analysis of carbon and hydrogen isotope could be performed in real time. Thus, a fiber optics-based gas sensing technique at mid-IR wavelengths was demonstrated, and we expect to develop fiber-optic sensors using the IR region by combining Bragg grating for fluoride fibers^21^.

## Conclusion

We have developed a 3-µm mid-IR ASE light source pumped by means of energy transfer from Er^3+^ to Dy^3+^ with a simple system consisting of Er^3+^/Dy^3+^ co-doped ZBLAN double-clad fiber. A broadband and moderate-power ASE light source of 2.5–3.7 µm was experimentally demonstrated by optimizing the design of the ASE light source in terms of ion concentration, fiber length, pump power, and pumping configuration. We have confirmed that the fabricated light source can be used for CH_4_ gas detection, indicating that it may also have other sensing applications. The wavelength broadening unexpectedly reached more than 3.7 µm with a full-width at half maximum of 1220 nm. By considering the discrepancy between obtained spectra of bulk ZBLAN glass and ZBLAN fiber co-doped with Er^3+^/Dy^3+^, the performance of the ASE light source can be improved by adjusting the concentration ratio. Our method can facilitate a simplified environmental monitoring system. Ongoing detailed research demonstrating multiple-gas sensing and fiber inline spectroscopy using a fluoride fiber-optic sensor with the potential for industrial application will be performed and reported in the future.
